# A *LEA* Gene Regulates Cadmium Tolerance by Mediating Physiological Responses

**DOI:** 10.3390/ijms13055468

**Published:** 2012-05-04

**Authors:** Caiqiu Gao, Chao Wang, Lei Zheng, Liuqiang Wang, Yucheng Wang

**Affiliations:** State Key Laboratory of Forest Genetics and Tree Breeding, Northeast Forestry University, 26 Hexing Road, Harbin 150040, China; E-Mails: chwcaogcq@yahoo.com.cn (C.G.); wzyrgm@163.com (C.W.); zhenglei123@126.com (L.Z.); liuqiangwang2009@yahoo.com (L.W.)

**Keywords:** LEA gene, cadmium stress, stress tolerance, gene transformation, physiological response

## Abstract

In this study, the function of a *LEA* gene (*TaLEA1*) from *Tamrix androssowii* in response to heavy metal stress was characterized. Time-course expression analyses showed that NaCl, ZnCl_2_, CuSO_4_, and CdCl_2_ considerably increased the expression levels of the *TaLEA1* gene, thereby suggesting that this gene plays a role in the responses to these test stressors. To analyze the heavy metal stress-tolerance mechanism regulated by *TaLEA1*, *TaLEA1*-overexpressing transgenic poplar plants (*Populus davidiana* Dode × *P. bollena* Lauche) were generated. Significant differences were not observed between the proline content of the transgenic and wild-type (WT) plants before and after CdCl_2_ stress. However, in comparison with the WT plants, the *TaLEA1*-transformed poplar plants had significantly higher superoxide dismutase (SOD) and peroxidase (POD) activities, and lower malondialdehyde (MDA) levels under CdCl_2_ stress. Further, the transgenic plants showed better growth than the WT plants did, indicating that *TaLEA1* provides tolerance to cadmium stress. These results suggest that *TaLEA1* confers tolerance to cadmium stress by enhancing reactive oxygen species (ROS)-scavenging ability and decreasing lipid peroxidation. Subcellular-localization analysis showed that the TaLEA1 protein was distributed in the cytoplasm and nucleus.

## 1. Introduction

During the late stages of seed development, newly synthesized proteins are typically involved in induction of desiccation tolerance [1]. Late embryogenesis abundant (LEA) proteins are members of a large group of hydrophilic proteins that accumulate during the late stages of seed development and are involved in the development of desiccation tolerance in maturing seeds. The LEA proteins are specifically supposed to be involved in mediating tolerance to osmotic stress. These proteins are widely distributed in the plant kingdom. Studies have shown that plant LEA proteins are encoded by multigene families [2]. The first LEA protein was isolated from wheat (*Triticum aestivum* L.) embryos and was described as an early methionine-labeled (Em) polypeptide [3].

The exact functions of LEA proteins are not known. However, studies have shown that LEA proteins can improve plant tolerance to salt, drought, and/or freezing stresses through various physiological pathways. For instance, LEA proteins can stabilize the membrane by inducing preferential hydration or replacing water during desiccation conditions [4]. LEA proteins can also act as molecular chaperones or shields that prevent irreversible protein aggregation during stress conditions [5]; this finding proves that LEA proteins can bind to nonnative proteins to prevent protein aggregation and maintain them in a folding-competent state. Exposure to adverse conditions often causes oxidative stress and reactive oxygen species (ROS) generation in plants. Therefore, an effective ROS-scavenging system is important for plants. LEA proteins can directly reduce oxidative stress by scavenging ROS and indirectly reduce ROS production by sequestering the metal ions that generate ROS in dehydrating cells [6]. Since LEA proteins are highly hydrophilic, they may also act as hydration buffers and decrease the water-loss rate during stress conditions. Soulages *et al*. [7] showed that LEA proteins can replace the water at the interaction interface with other macromolecules, thereby protecting cells or tissues from drought damage. In plant cells, dehydration can cause an increase in the concentration of ions, resulting in damage to the architecture and function of macromolecules. Because LEA proteins have many charged amino acid residues, they can play a role in sequestering ions during water loss [6].

Although the roles of the LEA proteins in mediating tolerance to abiotic stresses such as those caused by salt, drought, and low temperature, have been investigated, the role of LEA proteins in heavy metal stress has not been determined. Moreover, the physiological changes mediated by LEA proteins in response to heavy metal stress are still unknown.

*Tamarix androssowii* is a woody plant that is distributed in central Asia and China. *T. androssowii* can grow well in adverse environments, thereby indicating that it possesses molecular and physiological systems to adapt to salt stress. Therefore, *T. androssowii* is a valuable model to characterize the genes and mechanisms for stress tolerance in plants.

In this study, the expression of a *LEA* gene (*TaLEA1*) from *T. androssowii* was investigated in response to NaCl, ZnCl_2_, CuSO_4_, and CdCl_2_ stress. To understand the physiological *LEA*-mediated responses in plants during CdCl_2_ stress, *TaLEA1* was further introduced into poplar plants by using an *Agrobacterium*-mediated method. Stress-related physiological parameters were compared between the transgenic and wild-type (WT) plants, including peroxidase (POD) and superoxide dismutase (SOD) activities and the malondialdehyde (MDA) and proline content. Our results provide mechanistic details of the CdCl_2_ tolerance conferred by *LEA* in relation to the physiological changes observed.

## 2. Results

### 2.1. Time-Course Analysis of TaLEA1 Expression

**Figure 1:**
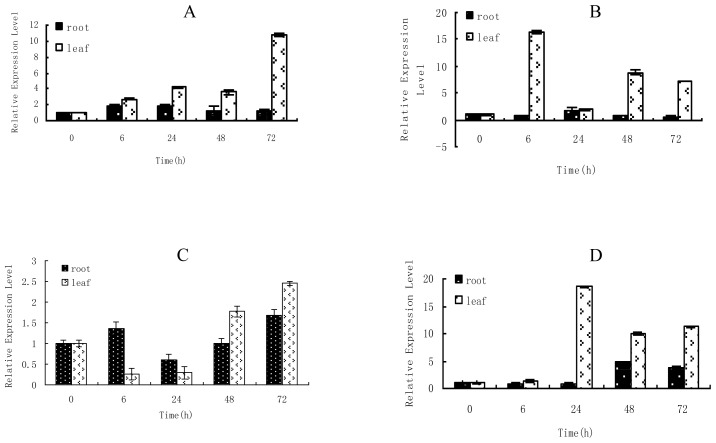
Time course analyses of the expression of *TaLEA1* in response to different abiotic stresses by using qRT-PCR. **A**: 0.4 M NaCl; **B**: 150 μM ZnCl_2_ stress; **C**: 150 μM CuSO_4_ stress; **D**: 150 μM CdCl_2_ stress. qRT-PCR data was normalized using α-tubulin and β-actin genes from *T. androssowii* and is shown relative to 0 h.

Real-time RT-PCR was performed to study the *TaLEA1* expression in response to NaCl, ZnCl_2_, CuSO_4_, and CdCl_2_. The results showed that the *TaLEA1* gene was expressed in both roots and leaves, and the expressions in roots and leaves were differently regulated by the stressors (Figure 1). NaCl stress induced high expression levels of *TaLEA1* in leaves; after 72 h of NaCl stress, the *TaLEA1* expression was 10.8-fold upregulated. However, NaCl stress did not induce considerable differences in the *TaLEA1* expression in roots. ZnCl_2_ stress induced high expression levels of *TaLEA1* in leaves, with the maximum expression (16.4-fold) after 6 h of stress; however, ZnCl_2_ stress did not cause considerable differences in *TaLEA1* expression in roots. CuSO_4_ stress did not cause considerable differences in the *TaLEA1* expression in roots. In leaves, CuSO_4_ transiently inhibited *TaLEA1* expression at 6 and 24 h after induction of stress; however, *TaLEA1* was highly upregulated at 48 and 72 h of CuSO_4_ stress. Under CdCl_2_ stress, the *TaLEA1* expression in roots was not differently regulated at 6 and 24 h of stress, but was up-regulated at 48 and 72 h of stress. In leaves, the *TaLEA1* expression was not differently regulated after 6 h of CdCl_2_ stress, but was highly up-regulated after 24–72 h of stress, with the expression peak (18.5-fold) after 48 h of stress.

### 2.2. Generation of Transgenic Poplar

**Figure 2:**
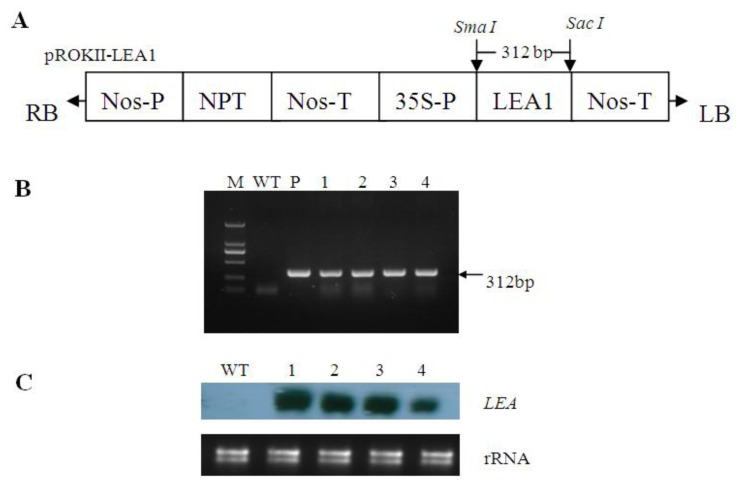
Molecular detection of the *TaLEA1* transformed poplar plants (*Populus davidiana* Dode ×*P. bollena* Lauche). A: Diagram of the T-DNA region of the vector pROKII–LEA used for transformation. 35S-P: CaMV 35S promoter, LEA: coding region of *TaLEA1* gene. B: Detection of the transgene from kanamycin-resistant lines by PCR; P positive control (pROKII–LEA), WT wild type plants, 1–4 independently *TaLEA1* transformed plant lines. C: Analysis of the expression of transgene in the transgenic lines by Northern blot, WT wild type plants, 1–4 independently *TaLEA1* transformed plant lines.

Four independent kanamycin-resistant poplar lines were generated using the *Agrobacterium*-mediated method. PCR was performed to confirm the transformation of heterologous *TaLEA1*. All transgenic lines showed the expected band and the WT samples did not show this band (Figure 2B), thereby confirming the integration of *TaLEA1* in the poplar genome. From Northern blot analysis, each transgenic line exhibited a distinct band, the length of which was consistent with the predicted length of *TaLEA* mRNA, while the WT lines did not produce this band. These data confirmed that the *TaLEA* gene was successfully integrated and expressed in the transgenic poplar lines (Figure 2).

### 2.3. Comparison of the Growth between TaLEA-Transformed and WT Poplar Plants after CdCl_2_ Exposure

**Figure 3:**
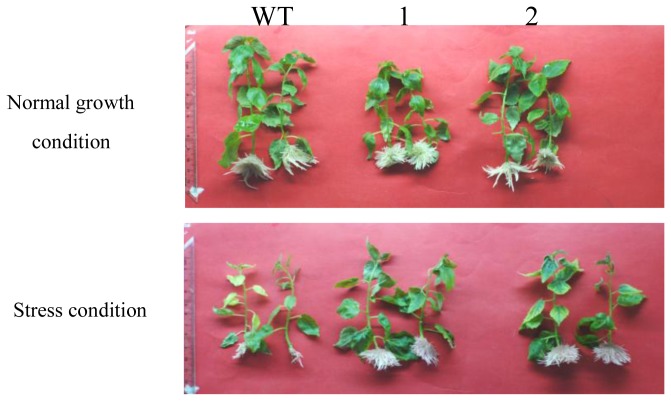
Comparison of growth between wild type and *TaLEA1* transformed plants. Two independent transgenic poplar lines and WT plants with similar heights (about 1 cm) were grown on half-strength MS rooting medium supplemented with 100 μM CdCl_2_, and photographed after 20 days. A: the transgenic and WT plants grown under normal growth condition; B: the transgenic and WT plants grown under 100 μM CdCl_2_ stress condition.

Transgenic and WT poplar plants were cultured in half-strength MS medium supplemented with 100 μM CdCl_2_. After 20 days, we compared the growth of the transgenic and WT plants, including their leaf length and root numbers (Figure 3; Table 1). All the transgenic plants exhibited much better growth (including longer leaf length and higher root numbers) than the WT plants did, thereby suggesting that the heavy metal tolerance of transgenic plants was higher than that of WT plants. Interestingly, although the root number in transgenic line 2 increased after stress, the roots formed after stress were thinner and shorter than those formed before stress (Figure 3).

### 2.4. Analyses of POD Activity in TaLEA1-Overexpressing Plants

**Figure 4:**
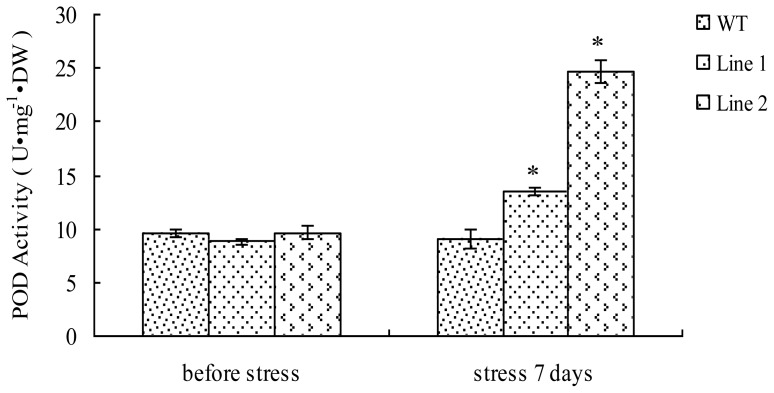
Peroxidase (POD) activity analysis of the *TaLEA1* transformed and WT poplar lines. POD activity was determined before stress and after 100 μM CdCl_2_ stress for 7 days. Values are expressed as means (*n* = 9 plants); error bars denote SD. * Significant (*t* test, *P* < 0.05) difference compared with WT plants.

We measured and compared the POD activities in the transgenic and WT plants before and after exposure to CdCl_2_ stress (Figure 4). There was no significant (*P* > 0.05) difference between the POD activities of transgenic and WT plants before CdCl_2_ stress treatment. However, after 7 days of stress, POD activities in both transgenic lines were significantly (*P* < 0.05) higher than that in WT plants. In particular, in transgenic line 2, the POD activity was 2.74-fold higher than that in WT plants.

### 2.5. Analysis of the SOD Activity in TaLEA1-Overexpressing Plants

**Figure 5:**
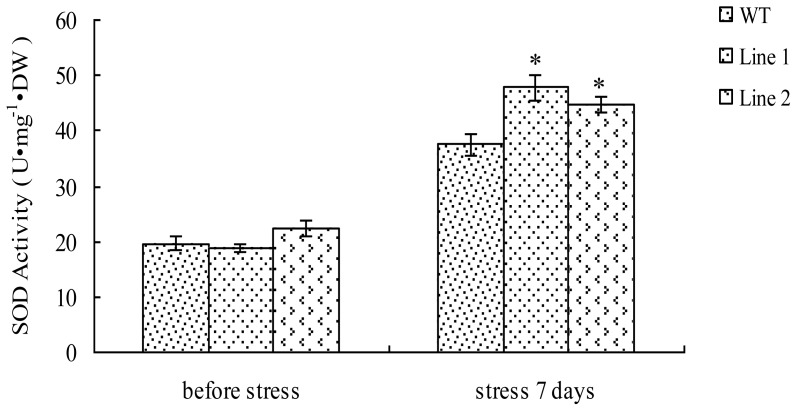
Analysis of superoxide dismutase (SOD) activity in the *TaLEA1* transformed and WT poplar lines. SOD activity was determined before stress and after 100 μM CdCl_2_ stress for 7 days. Values are expressed as means (*n* = 9 plants); error bars denote SD. * Significant (*t* test, *P* < 0.05) difference compared with WT plants.

There was no significant (*P* > 0.05) difference between the SOD activities of transgenic lines and WT plants prior to exposure to stress (Figure 5). The SOD activities in both transgenic and WT poplar plants were elevated under CdCl_2_ stress. However, the SOD activities in the two transgenic lines were 27.6% and 20%, respectively, higher than that in the WT plants (Figure 5). In addition, the SOD activity in both transgenic lines was significantly (*P* < 0.05) higher than that in WT plants under CdCl_2_ stress.

### 2.6. Analysis of MDA Content in TaLEA1-Overexpressing Plants

**Figure 6:**
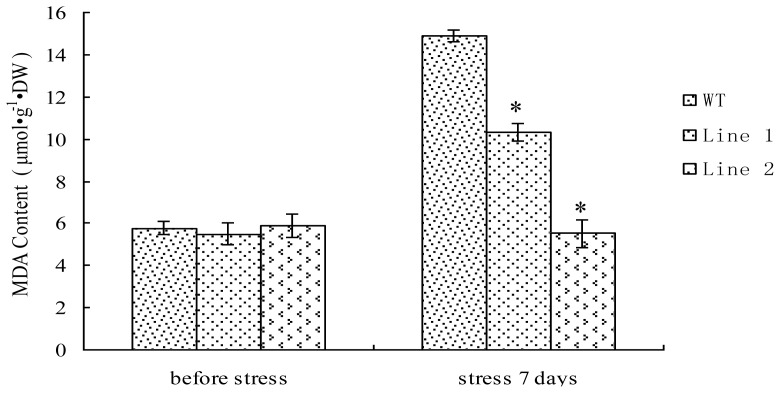
Analysis of malondialdehyde (MDA) level in the *TaLEA1* transformed and WT poplar lines. MDA level was determined before stress and after 100 μM CdCl_2_ stress for 7 days. Values are expressed as means (*n* = 9 plants); error bars denote SD. * Significant (*t* test, *P* < 0.05) difference compared with WT plants.

We investigated the influence of CdCl_2_ treatment on the MDA content in the transgenic and WT plants (Figure 6). The MDA content in the transgenic and WT plants did not differ significantly (*P* ≥ 0.05) prior to exposure to stress. The MDA content in WT plants considerably increased after CdCl_2_ stress for 7 days. However, in comparison with the WT plants, the transgenic plants did not show a substantial increase in the MDA content, especially transgenic line 2. Moreover, under CdCl_2_ stress, the MDA content of both transgenic lines was significantly (*P* < 0.05) lower than that in WT plants.

### 2.7. Analysis of the Proline Content in TaLEA1-Overexpressing Plants

**Figure 7:**
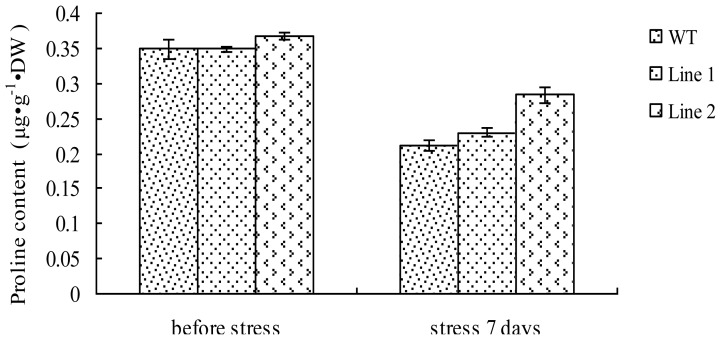
Proline content analysis of the *TaLEA1* transformed and WT poplar lines. Proline content was determined before stress and after 100 μM CdCl_2_ stress for 7 days. Values are expressed as means (*n* = 9 plants); error bars denote SD.

We analyzed the proline content in both transgenic and WT plants (Figure 7). Under CdCl_2_ stress, proline was inhibited in both WT and transgenic plants. There was no significant (*P* ≥ 0.05) difference between the proline content in transgenic and WT plants before and after CdCl_2_ stress. These results indicated that LEA overexpression did not affect proline accumulation.

### 2.8. Subcellular Localization of TaLEA1

**Figure 8:**
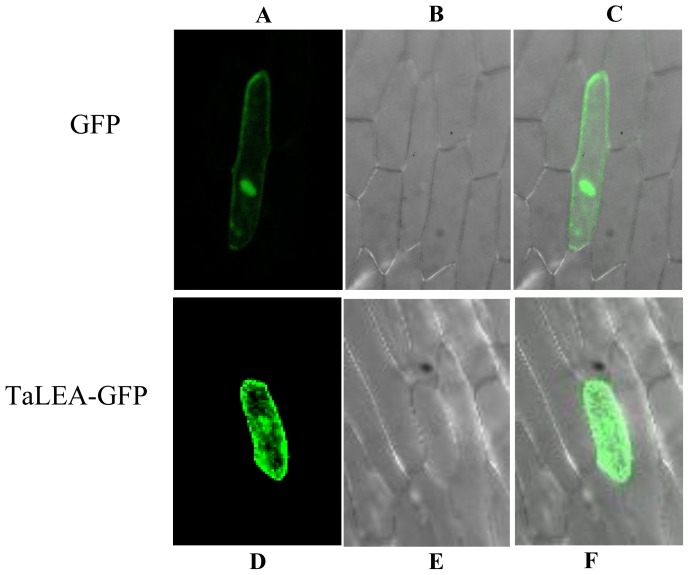
Subcellular location analysis of TaLEA1 transformed protein. The full-length coding region of *TaLEA1* (without the stop codon) was fused with the *N*-terminus of the GFP protein to generate the fusion gene *TaLEA1*-*GFP*. The fusion construct for TaLEA1-GFP and the GFP control plasmid were introduced into onion epidermal cells by particle bombardment. **A**, **D**: GFP fluorescence; **B**, **E**: onion peel cells imaged under bright field; **C**, **F**: merge of bright field and fluorescence.

Subcellular localization of *TaLEA1* was analyzed using a transient expression assay. Two constructs encoding a TaLEA-GFP or a GFP protein were introduced into onion epidermal cells by particle bombardment. The results showed that the control GFP protein was uniformly distributed throughout the cells. However, the TaLEA-GFP fusion protein was observed only in the cytoplasm and nucleus, indicating that the TaLEA1 protein is distributed in the cytoplasm and nucleus (Figure 8).

## 3. Discussion

*LEA* gene expression has been shown to be induced by dehydration, low temperature, salinity, and Abscisic Acid(ABA) exposure [3,8]. However, *LEA* gene expression in response to heavy metal stress has not been elucidated. In the present study, our results showed that the *TaLEA1* gene can be induced by heavy metal stress such as ZnCl_2_, CuSO_4_, and CdCl_2_, in particular ZnCl_2_ and CdCl_2_, thereby suggesting that the *TaLEA1* gene is also involved in the heavy metal response. Notably, *TaLEA1* induction in leaves was much higher than that in roots under all the stresses tested, including those induced by ZnCl_2_, CuSO_4_, NaCl, and CdCl_2_; this finding implies that the stress-resistance activity of the *TaLEA1* gene is primarily observed in the leaves rather than the roots.

*LEA* genes provide tolerance to various abiotic stresses, such as freezing, osmotic, and salt stresses. For instance, overexpression of *LEA* genes in transgenic plants was found to improve the plants’ resistance to salt, freezing, and osmotic stress [8–10]. However, there is little information on whether the *LEA* genes confer tolerance to heavy metal stresses. Gianazza *et al*. [11] studied protein expression in *Lepidium sativum* L. plantlets in response to Cd using two-dimensional electrophoresis combined with ESI-MS, and their results showed that two LEA proteins were induced by Cd stress. Our study showed that *TaLEA1* significantly improved CdCl_2_ tolerance in transgenic plants, thereby confirming that *LEA* genes also play a role in heavy metal tolerance. Therefore, LEA genes confer tolerance to various stresses.

Under adverse environments, plants usually show rapid generation of reactive oxygen species (ROS), and ROS ultimately induces secondary oxidative stress in plants [12]. Therefore, the ROS-scavenging capacity of plants should be improved to enhance their resistance to adverse stress conditions. SOD plays an important role in ROS scavenging in plants. In the present study, CdCl_2_ stress elevated SOD activity in both transgenic and WT plants. Obviously, the improved SOD activity is a plant response to CdCl_2_ stress. Our results showed that there was no difference between the SOD activities in transgenic and WT plants before exposure to CdCl_2_ stress. However, after exposure to CdCl_2_ stress, the SOD activity in the transgenic lines was at least 20% higher than that in the WT plants, thereby suggesting that LEA can confer stress tolerance by improving SOD activity under CdCl_2_ stress.

Plant PODs have been found to be involved in a variety of biological functions, such as hydrogen peroxide detoxification, stress responses, lignin biosynthesis, and hormone signaling [13]. PODs are a group of important antioxidant enzymes that play a protective role in ROS scavenging [14]. Plant POD genes are activated in response to abiotic stresses, and transgenic plants overexpressing heterologous POD genes show high levels of tolerance to abiotic stresses [15,16]. In the present study, our results showed no differences between the POD activities in the transgenic and WT plants before exposure to stress; however, under CdCl_2_ stress, the POD activity in the transgenic lines was significantly (*P* < 0.05) higher than that in the WT plants, thereby suggesting that *TaLEA1* overexpression can improve POD activity in transgenic plants. Therefore, one method by which *TaLEA1* enhances stress tolerance is by improving POD activity under CdCl_2_ stress.

Abiotic stresses result in excessive accumulation of ROS in plants, and the accumulated ROS cause lipid peroxidation in plants [17]. MDA is an end-product of lipid peroxidation in biomembranes and free radical chain reactions; therefore, the MDA content can represent the extent of lipid peroxidation and membrane injury. In the present study, under CdCl_2_ stress, the MDA levels in all the *TaLEA1*-transformed lines were significantly (*P* < 0.05) lower than in the WT plants (Figure 6), thereby indicating that lipid peroxidation in the transgenic plants was lower than that in the WT plants. Therefore, *TaLEA1* overexpression may contribute to reduce lipid peroxidation under salt stress. The reduced lipid peroxidation in the transgenic plants under CdCl_2_ stress may be attributed to the fact that *TaLEA1* overexpression enhanced the activity of the ROS-scavenging enzymes SOD and POD.

Proline is one of the most important osmolytes that accumulate in plants exposed to osmotic stress conditions. In plants, proline usually functions as an osmolyte for the intracellular osmotic adjustment; it also serves as a sink for the energy to regulate redox potentials, as a scavenger of hydroxyl radicals, and as a solute protecting macromolecules from denaturation [18,19]. Some studies [20] showed that increasing the proline level can significantly elevate plant tolerance to Cd stress. In the present study, our results showed that the proline content in both transgenic and WT plants reduced after exposure to CdCl_2_ stress for 7 days, and there was no significant (*P* > 0.05) difference between the proline levels in transgenic and WT plants before or after CdCl_2_ stress, thereby indicating that *LEA* gene overexpression was not involved in proline accumulation in plants.

## 4. Experimental Section

### 4.1. Plant Culture and Treatments

The seedlings of *T. androssowii* were grown in pots containing a mixture of turf peat and sand (2:1 *v*/*v*) under controlled greenhouse conditions: light intensity, 400 μmol·m^−2^·s^−1^, 65–75% relative humidity 14:10-h light-dark cycle, and average temperature of 24 °C. Well-watered 4-month-old seedlings were used as experimental material. To induce abiotic stresses, the seedlings were watered into their roots with solutions of 0.4 M NaCl, 150 μM ZnCl_2_, 150 μM CdCl_2_, or 150 μM CuSO_4_ for 0 (no treatment with the stress-causing agent, control), 6, 24, 48, and 72 h. After the treatments, leaves and roots from each sample (containing 10 seedlings) were harvested and pooled for real-time reverse transcription-polymerase chain reaction (RT-PCR) analyses.

### 4.2. Expression Analysis of TaLEA1 in Response to Different Stresses

*TaLEA1* (GenBank number, DQ663481, belongs to Lea 3 family), a *LEA* gene cloned from *T. androssowii* was used for expression analysis. Total RNA from each sample was reverse transcribed into cDNA by using oligo (deoxythymidine) primers with the PrimeScript™ RT reagent Kit (TaKaRa, China). Real-time RT-PCR was performed in an MJ Opticon 2 System (Bio-Rad, Hercules, CA, USA) with α-tubulin and β-actin genes as the reference genes. The primers used in real-time reverse transcription–PCR are shown in Table 2. Each reaction was performed with 3 replicates to ensure reproducibility of results. The expression levels were calculated from the cycle threshold according to the ΔΔCT method [21].

### 4.3. Plant Transformation

The complete open reading frame (ORF) of *TaLEA1* was inserted into the pROKII plant expression vector under the control of the CAMV-35S promoter (Figure 2A) and transferred into *Agrobacterium EHA105. TaLEA1* was introduced into poplar plants (*Populus davidiana* Dode *×P. bollena* Lauche) using an *Agrobacterium*-mediated method as described by Tzfira *et al*. [22] with little modification. Five kanamycin-resistant lines were generated. In PCR analysis of the transgenic plants, the following primers were used to amplify a characteristic 312-bp fragment of *TaLEA1:* forward primer, 5′-ATGGCTCGCTGCTCTTACTC-3′; reverse primer, 5′-TCAGTGAGAGGATCGATTGAAC-3′. For Northern blot analysis, probes were labeled with DIG-dUTP by PCR amplification using *LEA* forward and reverse primers. RNA (20 μg) was fractionated on a denaturing formaldehyde agarose gel, blotted on Hybond N^+^ membranes, and fixed by ultraviolet (UV) cross-linking (254 nm, 8 min). The membranes were prehybridized (68 °C, 2 h), hybridized with the probes (68 °C, 18 h), and detected according to the instructions provided in the manual (Dig Northern starter kit, Roche).

### 4.4. Growth Analysis of Transgenic and WT Poplar Cultured in CdCl_2_ Media

Two independent transgenic poplar lines and WT plants with similar heights (about 1 cm in length) were grown on half-strength Murashige & Skoog (MS) rooting medium (1/2 MS + 0.25 mg/L NAA + 2% sucrose) supplemented with 100 μM CdCl_2_. WT plants were used as negative controls. Plants were cultured at 25 °C with a 16-h photoperiod under artificial light. After 20 days, the growth levels of the transgenic and WT plants were compared. The experiment was repeated at least twice, with at least 20 seedlings for each line.

### 4.5. Determination of Heavy Metal Tolerance of the TaLEA1 Transgenic Poplar Plants

At least 20 plantlets for the 2 transformed lines and WT poplar were used for heavy metal tolerance analysis. The plantlets were well hydrated prior to exposure to 100 μM CdCl_2_ stress, and the following physiological and biochemical indices were measured at 0 and 7 days after exposure to the stress: SOD and POD activities, and MDA and proline content.

### 4.6. Measurement of the Physiological Parameters in Transgenic Poplar Plants

SOD and POD activities and MDA content were determined according to the method of Wang *et al*. [23]. Proline content was determined using the method by Bates *et al*. [24]. Each sample included 9 plantlets and the leaves were used to produce physiological parameters. Each experiment was performed in triplicate to ensure accuracy of analyses, and the error bars show standard deviation from the mean.

### 4.7. Subcellular Localization of the TaLEA Gene

For subcellular localization analysis, the *TaLEA1* coding region without the termination codon was ligated in frame to the *N*-terminal of the green fluorescent protein to generate the TaLEA-GFP fusion gene. A CaMV-35S promoter was used to drive TaLEA-GFP, and 35S-GFP was used as the control. The plasmids encoding the TaLEA-GFP fusion protein and the 35S-GFP control were introduced into onion epidermis cells by using particle bombardment (Bio-Rad). The transformed cells were observed using the confocal laser scanning microscope LSM410 (Zeiss, Jena, Germany) with the following settings: for GFP excitation at 488 nm and emission of 507 nm long pass.

### 4.8. Data Analyses

The data analyses were performed using SPSS version 11.5 (SPSS Inc., Chicago, IL, USA). Mean comparisons were performed using Tukey’s HSD test. The level of significance for all analyses was set at *P* ≤ 0.05. Sample variability was represented as the standard deviation (SD).

## 5. Conclusions

In summary, high degrees of *TaLEA1* gene expression can be induced by NaCl and heavy metal stressors, including ZnCl_2_, CuSO_4_, and CdCl_2_, thereby demonstrating the role of *LEA* genes in the stress responses to different metals. Under CdCl_2_ stress, *TaLEA1* overexpression considerably improved POD and SOD activity and decreased MDA content in plants. However, *TaLEA1* is not involved in proline accumulation under stress. The CdCl_2_ stress tolerance of *TaLEA1* transgenic plants was considerably higher than that of the WT plants, indicating that the *TaLEA1* gene may confer stress tolerance by enhancing ROS-scavenging ability. Therefore, the *TaLEA1* gene may be an excellent choice for a heavy metal tolerance gene in genetically engineered salt-tolerant plants.

## Figures and Tables

**Table 1:** Comparison of growth between *TaLEA1* transgenic and WT plants. The 35S::LEA lines and wild-type were grown in 1/2MS rooting medium with 100 μM CdCl_2_ for 20 days.

	Roots Number		Leaf Length (cm)	
	
Plants	Normal growth condition	Stress condition	Normal growth condition	Stress condition
WT	41 ± 0.52	11 ± 0.65	2.67 ± 0.13	1.65 ± 0.06
Line 1	47.5 ± 0.91 *	57 ± 1.03 *	2.39 ± 0.05	2.33 ± 0.01 *
Line 2	32.5 ± 0.84 *	56.5 ± 0.98 *	2.40 ± 0.08	2.43 ± 0.07 *

The root numbers and leaf length were measured under stress and normal growth condition. Values are the means ± S.D.

* Significant at 5% level compared with wild type plants (*t*-test).

**Table 2:** Primer sequences used for quantitative RT-PCR analysis.

Gene	Forward and Reverse Primers (5′–3′)
*TaLEA*	5-TAAATCACGAGGCGGCGAAACA-3 5-AACTCCAGCAACGTCATGCGAAGA-3
α-*actin*	5-AAACAATGGCTGATGCTG-3 5-ACAATACCGTGCTCAATAGG-3
*α-tubulin*	5-CACCCACCGTTGTTCCAG-3 5-ACCGTCGTCATCTTCACC-3
